# T cell subset profile in healthy Zambian adults at the University Teaching Hospital

**DOI:** 10.11604/pamj.2016.23.103.8547

**Published:** 2016-03-16

**Authors:** Caroline Cleopatra Chisenga, Paul Kelly

**Affiliations:** 1 Tropical Gastroenterology and Nutrition Group, University of Zambia School of Medicine, Lusaka, Zambia; 2 Blizard Institute Barts and The London School of Medicine, Queen Mary University of London, 4 Newark Street, London E1 2AT, UK and TROPGAN Group Department of Internal Medicine University of Zambia School of Medicine Nationalist Road, Lusaka, Zambia

**Keywords:** T cells, absolute CD4, CD8 count, HIV-seronegative

## Abstract

**Introduction:**

Symptom-free human immunodeficiency virus antibody-negative Zambian adults (51 subjects, aged 20 to 62 years, 33.3% women and 66.7% men) were studied to establish T cell subset reference ranges.

**Methods:**

We carried out across sectional study at the University Teaching Hospital, Lusaka. Blood samples were collected from healthy donor volunteers from hospital health care staff, between February and March 2015. Immunopheno typing was undertaken to characterize Tcell subsets using the markers CD3, CD4, CD8, α4β7, Ki67, CD25, CCR7, CD54RA, CD57, CD28, CD27 and HLA-DR.

**Results:**

Among 51 volunteers, Women had significantly higher absolute CD4 count (median 1042; IQR 864, 1270) than in men (671; 545, 899) (p=0.003). Women also had more CD4 cells expressing homing, naïve, effector and effector memory T cell subsets compared to men. However, in the CD8 population, only the effector cells were significantly different with women expressing more than the males.

**Conclusion:**

We provide early reference range for T cell subsets in Zambian adults and conclude that among the African women some T cell subsets are higher than men.

## Introduction

Many AIDS-related research studies are being conducted in Zambia and other African nations with a high prevalence of HIV infection. Common to these studies, is the measurements of total CD4 cell count, CD4 percentage and CD4/CD8 ratio in the peripheral blood. The enumeration of circulating CD4 and CD8 lymphocyte subsets is important because these cells are perturbed during HIV infection. Additionally, CD4 and CD8 measurements have been found to be useful surrogates for determining the risk of progression of HIV infection and are widely used in observational studies and AIDS clinical trials [1]. In low-income settings, HIV/AIDS related deaths are higher than in high-income settings [2]. One of the causes for this increased dearly mortality is having very low CD4 count at the start of antiretroviral therapy (ART) [3]. Studies conducted in Zambia have revealed that T cell subsets may be an accurate predictor of early mortality in HIV-infected adults [4] as well as in children [5] starting ART. However, there are no comparative data especially on normal parameters for T cell subsets in healthy adults. Several studies have evaluated CD4 and CD8 lymphocyte subsets in children [6–9] and adults in Western nations [10–15] and other countries [16–19]. However, data on normal ranges of CD4 and CD8 T cell subsets in Zambia are generally lacking. Thus, because of the lack of normal reference ranges for CD4 and CD8 T cell subset parameters in healthy African subjects, many investigators interpret their data using values that have been derived from populations in Europe and the United States. It would therefore be helpful to establish appropriate normal reference values for T cell subsets especially in African populations. In this study, we describe CD4 and CD8T cell subset reference ranges obtained by studying 51 symptom-free HIV-seronegative Zambians.

## Methods

**Study participants and area**: Participants were identified amongst staff of the University Teaching Hospital (UTH) between February and March 2015. Participants gave written informed consent. A total of 52 healthy volunteers consented and were recruited. Of the 52 participants; 20 were laboratory scientists, 22 medical students, 4 data clerks in the out-patient department, 1 nurse and the rest in administration (accountants).

**Ethical approval**: The University of Zambia Biomedical Research Ethics Committee approved the study (Ref. No. 009-01-11).

**Participant selection**: Inclusion criteria were at least 18 to 65 yrs old (either male or female), in good health, with normal full blood count parameters and of normal nutritional status. Exclusion criteria were HIV seropositive, HBsAg seropositive, hepatitis C virus seropositive, syphilis seropositive, participating in another conflicting study, pregnancy or having any chronic disease. All donors were evaluated for height, weight and grip strength using a grip strength dynamometer (Takei, Japan city)

**Qualitative testing HIV-1/HIV-2, HCV and HBsAg**: Following consent, 10 mls blood were collected into three EDTA tubes for haematology testing in the UTH haematology laboratory, CD4 and CD8 testing in the UTH virology laboratory and for HIV, syphilis, HBsAg and anti-HCV testing by ELISA. All the blood samples were delivered to the laboratory within 1 hour of collection. The ARCHITECT System Abbott i2000 (Abbott, Germany) was used to qualitatively detect HIV p24 antigen and antibodies to HIV-1/HIV-2, antibodies to anti-HCV, HBsAg and syphilis in plasma. The ARCHITECT qualitative assay is a chemiluminescent microparticle immunoassay (CMIA) for the qualitative detection of antibodies/antigens in serum or plasma. The presence or absence of antibodies or antigens in the sample was determined by comparing the chemiluminescent signal in the reaction to the cut-off signal, which was determined from an active calibration. Samples with a chemiluminescent signal greater than or equal to the cut-off signal were considered as reactive.

**Haematological analysis**: Haemoglobin measurements were undertaken immediately on whole blood on a Sysmex xt 4000i automated haematology analyser (Sysmex Corporation, Kobe, Japan).

**Total CD4 and CD8 estimation**: To measure total CD4 and CD8 counts, we used a FACSCaliber flow cytometer (Becton Dickinson, San Jose, USA) with MultiSET V2.2 software. CD3/CD4/CD45 TruC v2.0 (BD Biosciences) were used, and 15000 events were acquired. Antibodies used were conjugated to the following fluorochromes as follows: CD45-PerCP CD4-PE CD3-FITC.

**CD4^+^ and CD8^+^ T cell subset testing (immunophenotyping)**: To measure subsets, 100 µL of whole blood was stained for a wide range of markers: CD3, CD4, CD8, α4β7 integrin(gut homing panel), Ki67 (Proliferating panel), CCR7 and CD54RA (differentiation panel), CD57 (senescent panel), CD28, CD27 and HLA-DRor CD25 (activation panel). Cells were fixed, washed, and resuspended in 250 µL par formaldehyde or 250 µL BD Cell Fix diluted according the manufacturer's (BD Biosciences) instructions and 50-100,000 lymphocytes acquired on a FACS verse flow cytometer (Becton Dickinson, San Jose, USA). Intracellular Ki-67 staining was carried out following nuclear membrane permeabilisation (BD Perm/Wash ™ buffer from Becton Dickinson, San Jose, and U.S.A). Unstained cells and fluorescence minus one (FMO) controls were used to set all gates. Analysis of markers was carried out using BD FACSuite ™ software v1.0.2.

**Data analysis**: Data were entered and analysed using STATA software version 13 (StataCorp LP, College Station, Texas, USA). T cell markers were determined to be non-parametrically distributed using Shapiro-Wilk W test for normality, so data are presented as medians and inter quartile ranges and variables compared using the Mann-Witney U test. The χ^2^ test was used for testing associations of categorical variables. P values less than 0.05 were considered statistically significant. Reference intervals were derived from 2.5^th^ and 97.5^th^centiles for that variable.

## Results

### Characteristics of study participants

There were more men than women among the staff members recruited to this study, and the majority of these were in formal employment with a median age of 29 (IQR, 25-37) years (Table 1). Haemoglobin, body mass index (BMI) and grip strength results for both male and female participants were within international normal ranges. As expected, grip strength differed substantially by sex. Median total CD4 count was 824 (IQR, 558-1044) cells/µL and median CD8 count was 1371 (IQR, 1136-771) cells/µL. Most of the individuals were married and had attained university education.

**Table 1:** Baseline characteristics for the participants

Variable	Female N=17	Male N=34	p value
Sex	17 (33%)	34 (67%)	<0.001
Age (Years)	34 (27-45)	23 (28-34)	0.01
Absolute CD4 count (Cells/µL)	1042 (864-1270)	671 (545-899)	0.003
Absolute CD8 count (Cells/µL)	1531 (1248-1740)	1346 (1101-1801)	0.42
Hb (g/dl)	13.3 (11.3-14)	15.6 (14.8-16.6)	0.0001
BMI (Kg/m^2^)	25.03 (23.3-28.88)	23.18 (21.18-25.5)	0.07
Grip strength (Kg)	25.9 (23.8-27.5)	44.7 (39.9-47.7)	0.0001
**Education**			0.21
University	8 (47%)	22 (64%)	
College	6 (35%)	6 (18%)	
Secondary	2 (12%)	6 (18%)	
Primary	1 (6%)	0	
**Occupation**			0.22
Employed	11 (64%)	23 (68%)	
Students	4 (24%)	11 (32%)	
Business	1 (6%)	0	
Unemployed	1 (6%)	0	
**Marital status**			0.23
Married	10 (59%)	17 (50%)	
Single	6 (35%)	17 (50%)	
Divorce	1 (6%)	0	

**^1^**Mann–Whitney U-tests were used for non-parametric variables and Pearson chi square for categorical variables. Continuous characteristics are expressed as median (interquartile range) and dichotomous characteristics are expressed as n (%). g/dl=grams per decilitre; Hb= haemoglobin; BMI= body mass index

### CD4^+^and CD8^+^T cell subset distribution in healthy adult volunteers

**Figure 1:**
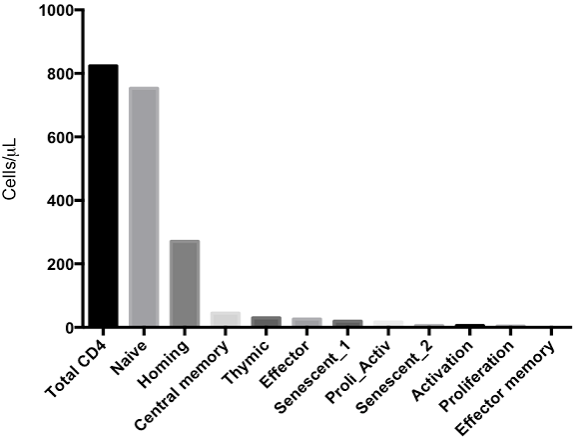
Total CD4 and subset distribution in healthy Zambian adults using surface marker expression

**Figure 2:**
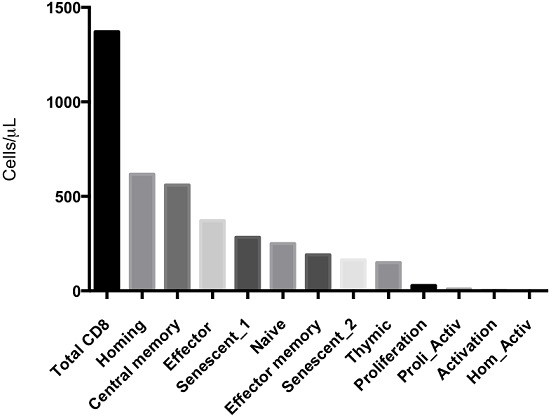
Shows total CD8 and subset distribution in healthy adults using surface marker expression

Non-viable (CD4^+^7AAD^+^) CD4+ T cells were completely absent in the healthy participants (data not shown).Figure 1 shows that majority of the CD4+ T cells in healthy adults were naïve (CCR7^+^CD45RA^+^) cells (median 753; IQR, 418-885 cells/µL), followed by recent thymic emigrant (CD31+CD45RA+) cells (median 30; IQR 22-35), gut homing cells (α4^+^β7^+^) 271 (IQR, 198-408) and gut homing and activated (α4^+^β7^+^CD25^+^) CD4^+^ T cells 170 (IQR, 129-269). In the CD8^+^ T cell subsets (Figure 2), there were no homing activated cells. Generally, cells expressing activation markers were minimal (2; 0-6), as were cells expressing proliferation markers (28;17-38), non-viable cells (zero) and cells expressing both proliferation and activation markers (10; 4-19). CD8+cells included recent thymic emigrants (149, 105-381), central memory (191, 109-282), senescent 2 (CD57^+^CD28^−^) cells (164, 36-332) and naïve CD8^+^ T cells (255, 127-401). Thus, in the CD8^+^ T cells population, the majority of cells are gut homing cells (617, 429-985) followed by effect or cells (564, 305-732).

**CD4 and CD8 subset reference intervals in healthy adults differ by sex:** stratification by sex showed that females had higher total CD4^+^ count (p=0.003), activated gut homing (p=0.01), naïve (p=0.02), effect or memory (p=0.01) and effect or (p=0.01) CD4^+^ T cells than males (Table 2). Where as in the CD8^+^population, only effect or cells were significantly different (p=0.03) between the females and males (Table 2). However, although females had higher CD4 and CD8 cells than the males, the overall distribution of both CD4 and CD8 was similar.

**Table 2:** CD4 and CD8 subset reference intervals in healthy population stratified by sex

Variable	Female N=17	Male N=34	
CD4	Median (95% RI)	Median (95% RI)	P value
Homing	413 (168-1134)	239 (17-673)	0.07
Homing activated	247 (89-628)	161 (21-413)	0.01
Thymic	31 (19-45)	28 (9-55)	0.21
Senescent 1	30 (1-191)	18 (5-278)	0.63
Senescent 2	10 (0-156)	6 (0-133)	0.46
Naïve	904 (184-1435)	573 (64-1283)	0.02
Central memory	51 (4-309)	43 (4-478)	0.72
Effector memory	1 (0-48)	0 (0-56)	0.01
Effector	55 (0-497)	9 (0-270)	0.01
Proliferating	22 (3-106)	12 (0-59)	0.30
Activated	4 (0-29)	7 (0-49)	0.85
Proliferating activated	3 (1-15)	4 (0-36)	0.10
**CD8**			
Homing	537 (11-1488)	627 (140-1436)	0.33
Homing activated	0 (0-0)	0 (0-4)	0.21
Thymic	146 (1-724)	38 (4-254)	0.47
Senescent 1	426 (3-700)	283 (69-1136)	0.99
Senescent 2	193 (0-691)	163 (0-1125)	0.47
Naïve	227 (3-754)	256 (60-1579)	0.29
Central memory	154 (2-494)	204 (37-1145)	0.23
Effector memory	435 (0-981)	316 (0-890)	0.08
Effector	695 (0-934)	472 (0-999)	0.03
Proliferating	28 (1-73)	29 (0-150)	0.91
Activated	4 (0-21)	14 (0-41)	0.47
Proliferating activated	1 (0-12)	2 (0-1281)	0.07

Values are medians, 95 percentiles are in parentheses. RI=Reference interval

## Discussion

In order to generate a reference group, a six colour FACSVerse instrument was used to characterise the CD4^+^and CD8^+^ T cells in HIV-negative adults in a Zambian population. Overall, we found more CD8 cells than CD4 cells (ratio of 1.67) in our Zambian population, contrary to what has been reported in other African studies and in Europe [20, 21]. With men and women taken together, the mean (SD) CD4^+^ cell count was 828 (336) cells/µL, which is higher than the values reported for healthy adult Ethiopians [22]. The values were comparable to those in Tanzanians [23] and lower than those reported for Ugandans [24].

Our data show that in healthy adults the majority of the CD4^+^ T cells were naïve. This finding was similar to findings from a study conducted in a similar cohort in San Francisco USA [25]. However, results for the healthy Ethiopians and Dutch showed lower numbers of naïve CD4^+^ T cells [26] compared to what was found in the Zambian cohort. When naïve CD4^+^ T cells are stimulated by their cognate antigen presented by competent antigen-presenting cells they differentiate into specialized effect or and/or memory cells. Effect or T cells induce cytolysis of infected cells or activate other lymphocytes and immune effect or cells, whereas memory T cells maintain the capacity to respond rapidly to previously encountered antigens [27]. In the healthy volunteers, however, there were very few circulating effector and or memory CD4^+^ T cells in the blood compared to effector and effector memory CD8^+^ T cells. Proliferating, proliferating and activated, activated and senescent CD4^+^ T cells in the healthy volunteers were very few. Since activation is triggered by the presence of antigen and is followed by proliferation, having few proliferating and activated cells may indicate absence of infectious disease and good T cell activation regulation in our healthy volunteers [28]. Furthermore, during infection, persistent T cell activation drives proliferation that results in the accumulation of senescent, antigen-experienced memory T cells with reduced expression of CD28 and increased expression of CD57 [29]. We found a higher number of senescent CD8^+^ T cells in the HIV-negative cohorts. Expression of CD 57 has been linked to greater resistance to apoptosis in CD8^+^ T lymphocytes especially during HIV-infection, facilitating accumulation [30]. Since CD8^+^ T cells are crucial to the recognition and clearance of virus-infected cells [31] a high number of senescent CD8^+^ T cells may compromise the ability of T cell immunity to suppress viral infections adequately. We also found a high number of CD4^+^ T cells expressing gut homing markers (about 35%) and activated gut homing (23%) in these healthy adults. In the CD8+population, approximately 39% were expressing gut homing cells. Gut homing cells are cells that are programmed to migrate to gut associated lymphoid tissues especially when there is an infectious challenge to the gut [32]. Finally, there were significant differences in absolute CD4 count between females and males in some of the subsets. The reference range of CD4 count was significantly higher in females than in the males, which is in agreement with other studies in Africa [33, 21]. We do not know whether this difference is due to environmental or genetic factors.

Our study has several limitations. All subjects were bled only once; therefore, it is possible that some subjects were in the “window” period of HIV infection, having been recently infected with HIV but not yet having developed anti-HIV antibodies. We did not perform medical or other laboratory examinations to evaluate the subjects′ general state of health but relied on self-reported symptom histories which may be inaccurate in excluding disease in individuals presenting for voluntary HIV screening. Therefore, the parameters used to describe the healthy volunteers were not exhaustive and as such, there could be other chronic viral infections such as cytomegalovirus or Epstein-Barr virus, which could have affected the CD4 and CD8 results. CD4 counts have been found to have significant diurnal and day-to-day variation in the same subjects and to vary with storage time and temperature [34]. While all blood samples were obtained during the late morning, stored at room temperature, and processed within a maximum of 2h after phlebotomy to minimize variability due to time of day and storage time and temperature among different subjects, we performed only single determinations of absolute CD4 count, and absolute CD8 count for each subject and cannot, therefore, comment on variability due to day-to-day variation among individuals. Furthermore, the sample size was small and samples were drawn from the same area.

## Conclusion

We propose that these reference intervals may be a useful point of comparison for studies on the immune system in Africa.

### What is known about this topic

- HIV perturbs CD4 and CD8 proportions
- Variations exist in proportion distribution between countries and lastly that differences between males and females exist in terms of T cell distribution

### What this study adds

- In Zambia, sex has an influence on T cell distribution
- Both CD4 and CD8 T cells are higher in females than males
- Because of this marked differences in the T cells, research linking changes in specific cellular subsets with specific disease conditions may aid in identifying HIV-infected adult males that may require more frequent clinical monitoring after starting ART compared to women
